# Concave Pit-Containing Scaffold Surfaces Improve Stem Cell-Derived Osteoblast Performance and Lead to Significant Bone Tissue Formation

**DOI:** 10.1371/journal.pone.0000496

**Published:** 2007-06-06

**Authors:** Antonio Graziano, Riccardo d'Aquino, Maria Gabriella Cusella-De Angelis, Gregorio Laino, Adriano Piattelli, Maurizio Pacifici, Alfredo De Rosa, Gianpaolo Papaccio

**Affiliations:** 1 Dipartimento di Medicina Sperimentale, Sezione di Istologia ed Embriologia, Secondo Ateneo di Napoli, Naples, Italy; 2 Dipartimento di Discipline Odontostomatologiche, Ortodontiche e Chirurgiche, Secondo Ateneo di Napoli, Naples, Italy; 3 Dipartimento di Medicina Sperimentale, Sezione di Anatomia Umana, Università degli Studi di Pavia, Pavia, Italy; 4 Dipartimento di Scienze Odontostomatologiche, Università degli Studi “G. d'Annunzio”, Chieti, Italy; 5 Department of Orthopaedic Surgery, Thomas Jefferson University, Philadelphia, Pennsylvania, United States of America; City of Hope Medical Center, United States of America

## Abstract

**Background:**

Scaffold surface features are thought to be important regulators of stem cell performance and endurance in tissue engineering applications, but details about these fundamental aspects of stem cell biology remain largely unclear.

**Methodology and Findings:**

In the present study, smooth clinical-grade lactide-coglyolic acid 85:15 (PLGA) scaffolds were carved as membranes and treated with NMP (N-metil-pyrrolidone) to create controlled subtractive pits or microcavities. Scanning electron and confocal microscopy revealed that the NMP-treated membranes contained: (i) large microcavities of 80–120 µm in diameter and 40–100 µm in depth, which we termed primary; and (ii) smaller microcavities of 10–20 µm in diameter and 3–10 µm in depth located within the primary cavities, which we termed secondary. We asked whether a microcavity-rich scaffold had distinct bone-forming capabilities compared to a smooth one. To do so, mesenchymal stem cells derived from human dental pulp were seeded onto the two types of scaffold and monitored over time for cytoarchitectural characteristics, differentiation status and production of important factors, including bone morphogenetic protein-2 (BMP-2) and vascular endothelial growth factor (VEGF). We found that the microcavity-rich scaffold enhanced cell adhesion: the cells created intimate contact with secondary microcavities and were polarized. These cytological responses were not seen with the smooth-surface scaffold. Moreover, cells on the microcavity-rich scaffold released larger amounts of BMP-2 and VEGF into the culture medium and expressed higher alkaline phosphatase activity. When this type of scaffold was transplanted into rats, superior bone formation was elicited compared to cells seeded on the smooth scaffold.

**Conclusion:**

In conclusion, surface microcavities appear to support a more vigorous osteogenic response of stem cells and should be used in the design of therapeutic substrates to improve bone repair and bioengineering applications in the future.

## Introduction

Engineering techniques capable of maximizing cell adhesion, performance and differentiation onto suitable scaffolds are needed to achieve and implement effective and efficient tissue reconstruction protocols. Polymer surface texturing and microstructures and physiological treatments with physical stimuli are found to ameliorate cell adhesion and differentiation and the overall tissue regeneration process [1], [2], [3]. However, clinical application of these bioengineering approaches and procedures is likely to require further and detailed understanding of complex interplays and interactions likely occurring at the cell surface/substrate interface. These interactions and interplays are likely to be mediated and affected by specific cell-surface components and submicroscopic scaffold features including microtextures. An example of microtextures are surface depressions or micro concavities of different dimensions which are found to be potentially important for stem cell differentiation [3]. It is possible that the specific size, conformation and distribution of these microcavities could affect the physiologic responses of stem cells within the scaffolds, for example by enhancing membrane contacts and cell surface exchanges; this could in turn direct their differentiation potentials along given lineages, ultimately leading to superior tissue production and repair [4]–[6]. Thus, it would be highly desirable to have a detailed understanding of stem cell/microcavity biology since it could lead to improved design of tissue-specific bioengineering applications.

In any bioengineering application, the selection of the stem cell population to be used is a critical factor. This is because stem cells of different origin have distinct capabilities in terms of survival, differentiation potentials, responses to experimental manipulations, and ultimately performance and effectiveness in tissue reconstruction. In previous studies, we have found that stromal cells isolated from adult human dental pulp (SBP-DPSCs) are multipotential and can give rise to a variety of cell types and tissues including adipocytes, neural cell progenitors and myotubes [7]–[9]. The cells proliferate extensively under standard culture conditions, have a long life-span, and maintain their multipotential capabilities for generations. When used under specific conditions, they can produce a 3D woven-bone tissue; upon transplantation *in vivo*, the tissue is actually remodeled to form a lamellar bone [7]–[8] through co-differentiation of SBP-DPSC into osteoblasts and endotheliocytes [10]. In this setting, SBP-DPSCs produce bone and not dentin as showed by in vitro mRNA transcripts, their high expression of alkaline phosphatase [7]–[8] – an enzyme that plays a pivotal role in bone mineralization [11] - and in vivo histomorphometry [10]. Therefore, these cells appear to be ideal candidates for bone-tissue reconstruction protocols and bone regeneration models.

Numerous scaffold materials, compositions and designs have been experimented and compared over recent years [12]. Amongst them, PLGA polymers remain popular as they display excellent biocompatibility demonstrated by absence of rejection and inflammation [13], [14]. Studies on toxicity, biocompatibility and clinical applications in the field of orthopedics and surgery, using implants made of polylactic acid and their copolymers, show that the intrinsic nature of these biomaterials renders them suitable for applications where temporary slow release of bioactive agents *in situ* are required [15]. The studies suggest also that these biomaterials are suitable for orthopaedic applications. In addition to biocompatibility [16], the other properties of these polymers that make them uniquely suitable for these and other applications include: thermoplasticity, high strength, controlled crystallinity, controlled degradation rates, controlled hydrophilicity, and proven non toxicity [17].

In bioengineering applications, bone formation is routinely monitored by a variety of histological, biochemical and molecular criteria. Chief among the latter is gene expression of growth factors [18] that are of particular importance to osteogenesis and bone growth and remodeling. Bone morphogenetic proteins (BMPs) are members of the transforming growth factor beta (TGF-β) superfamily and have long been known to be of significant importance for cartilage and bone differentiation during endochondral or intramembranous ossification [19]. BMP-2 is a well known effector of skeletal development and growth [18] and is also widely used for therapeutic bone reconstruction applications [18]. With respect to other BMPs, BMP-2 and BMP-4 are 92% identical at the amino acid level and are, therefore, considered a subgroup within the BMP family [19]. BMPs signal via different hetero-oligomeric complexes of type I and type II serine/threonine kinase receptors [20], [21]. BMP-2 receptors include the type I receptors, ALK-6/BMPR-IB, ALK-2/Act RI and ALK-3/BMPR-IA, and the type II receptors, BMP RII and Act RIIB [22]–[25]. During endochondral development, cartilage and bone differentiation involve a series of events that are directly influenced by BMPs. Endochondral bone formation is not only necessary for limb formation in embryogenesis, but is also required for longitudinal bone growth in postnatal life and bone regeneration following injury. BMP-2 is expressed in the growth plate and regulates growth plate chondrogenesis by inducing chondrocyte proliferation and hypertrophy [26], [27].

Vascular endothelial growth factor (VEGF) [28], also known as vascular permeability factor (VPF) [29] or vasculotropin [30], is a homodimeric 34–42 kDa, heparin-binding glycoprotein with potent angiogenic, mitogenic and vascular permeability-enhancing activities specific for endothelial cells. The expression of VEGF is upregulated by phorbol ester, TGF-β and in hypoxia [31]–[33]. Two receptor tyrosine kinases have been described as putative VEGF receptors. Flt-1 (fms-like tyrosine kinase) [34], and KDR (kinase-insert-domain-containing receptor) proteins have been shown to bind VEGF with high affinity [35]. Flk-1 has been shown to be involved in the VEGF-mediated transduction of signals that are important for angiogenesis and vasculogenesis [36]. *In vitro*, VEGF is a potent endothelial cell mitogen [28]–[30]. VEGF has also been shown to be chemotactic for monocytes and osteoblasts [37]. *In vivo*, VEGF can induce angiogenesis and increases microvascular permeability [28], [33], [37].

The present study was thus conducted to test whether the osteogenic potentials of SBP-DPSCs would be affected by the physical characteristics of PLGA-based substrates. Given the presumed importance of microcavities, we prepared substrates that were either smooth or contained microcavities of different dimensions. We monitored a variety of cellular parameters over time as well as the histomorphometric characteristics of bone and production of BMP-2 and VEGF, two important bone-related growth factors.

## Results

### Cell/scaffold interactions

As in previous studies from our laboratories, we isolated stromal stem cells from the dental pulps (SBP-DPSCs) of healthy individuals and sorted them to be c-Kit^+^/CD34^+^. After dental pulp extraction and digestion, almost 82.3±0.9% of cells obtained were viable.

The cells were grown under conditions favoring their commitment to the osteogenic lineage. By day 30 of culture the cells had become Runx-2 and CD44 double-positive by cytofluorimetric analysis as shown previously [7]–[9] and were also positive for Flk-1, VEGF receptor type II [10] and BMP receptors type I and II (data not shown).

**Figure 1:**
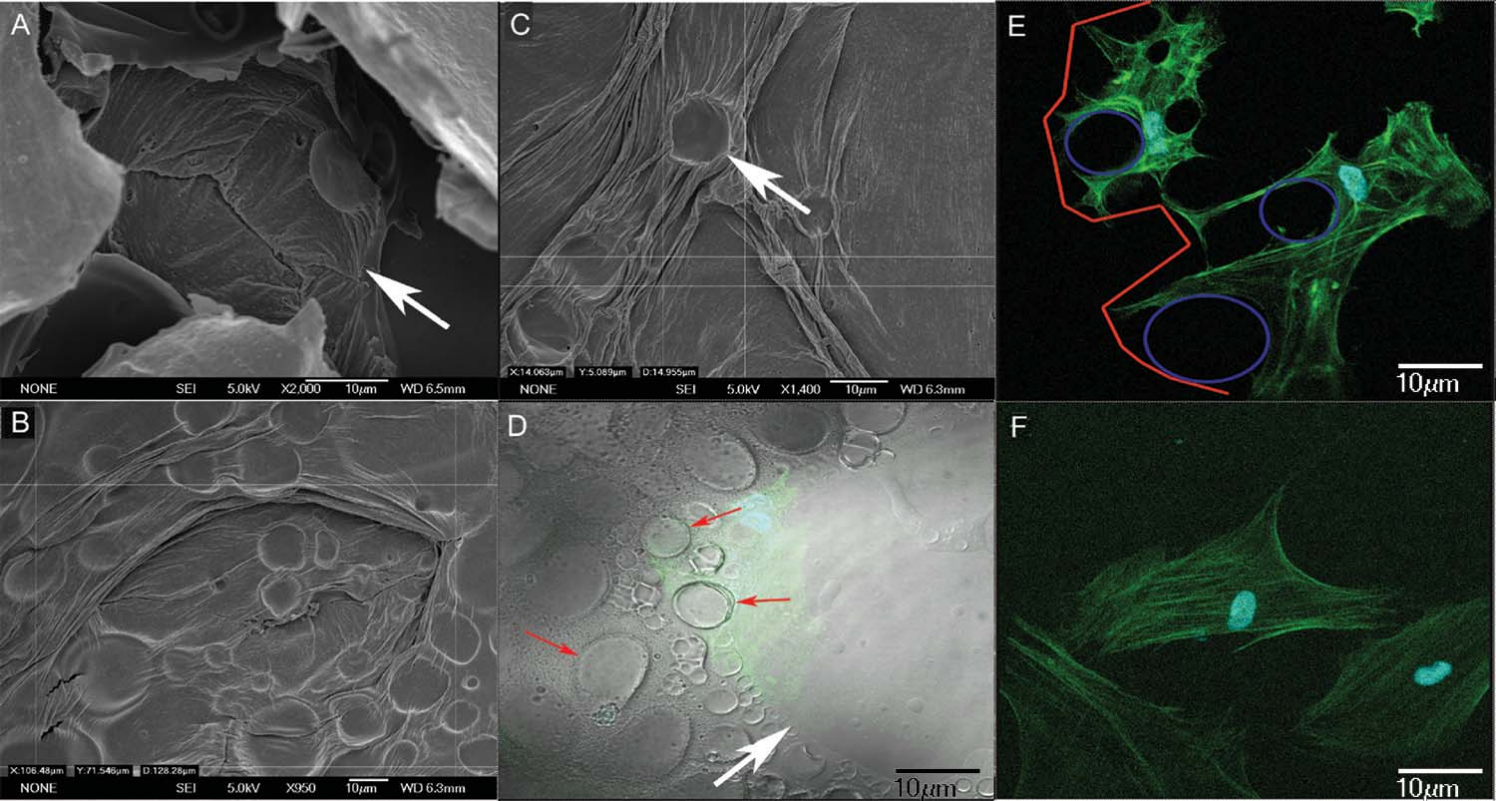
Images showing primary and secondary micro concavities at scanning electron and confocal microscopy. (**A**) Primary micro concavity (arrow) of the PLGA surface at SEM. Cells can be completely contained within a primary concavity, due to its dimensions. (Calibration Bar = 10 µm); (**B**) SEM analysis of primary concavity dimensions (Calibration Bar = 10 µm); (**C**) SEM analysis of secondary concavity dimensions (Calibration Bar = 10 µm); (**D**) The interaction between the concave surface, showing primary (white arrow) and secondary (red arrows) micro-concavities at the confocal microscope (in green a cell within a concavity). The intimate adherence of a cell to the polymer surface and its nuclear polarity are clearly observable. The image was been obtained superimposing dark field with light field confocal microscopy (Calibration Bar = 10 µm); (**E**) Confocal image showing primary (outlined in red) and secondary (outlined in blue) micro-concavities and spider-shaped cellular elongations (Calibration Bar = 10 µm); (**F**) A gingival fibroblast not showing cellular alterations or nuclear polarity at the confocal microscope (Calibration Bar = 10 µm).

**Figure 2:**
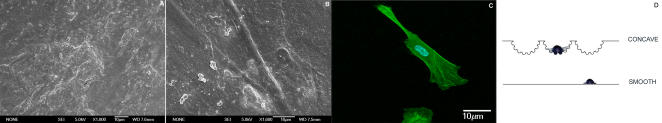
Images showing the results obtained when cells were cultured on the smooth surface. (**A**) smooth surface at SEM (Calibration Bar = 10 µm); (**B**) cells, cultured on a smooth surface, under SEM, show an elongated shape (Calibration Bar = 10 µm); (**C**) confocal image showing the relationship between cells and the smooth surface (Calibration Bar = 10 µm); (**D**) image showing the relationship between cells and the different surface texturing: (1) concave and (2) smooth.

Cells were then seeded onto PLGA 85:15 scaffolds. Scanning electron and confocal microscopy revealed that NMP (N-methyl-pyrrolidone)-treated scaffolds contained two types of concave microcavities: (i) large microcavities 80–120 µm in diameter and 40–100 µm in depth which we termed “primary” (Fig. 1A,B, *red arrows*); and (ii) smaller microcavities 10–20 µm in diameter and from 3–10 µm in depth, located within the primary cavities and thus termed “secondary” (Fig. 1A,B, *red arrowheads*) (see Fig 2D for schematic representation of distribution of primary and secondary microcavities). Membranes that were not treated with NMP had their original smooth surface characteristics (not shown). Cell plating efficiency tests showed that about 90% of osteogenic SBP-DPSC cells adhered to microcavity-containing NMP-treated surfaces as well as to culture flasks within 12 hrs, while about 80% adhered to smooth untreated surfaces. The approximate division time was about 2.4 days and it was the same both for cells grown in culture flasks and for cells on the scaffolds.

Given their large dimensions, primary microcavities were able to fully contain cells as shown by scanning and confocal microscopy (Fig. 1A,D,E, *green arrows*). The cells usually appeared to adhere and spread on the secondary microcavities, covering one or more of them (Fig. 1E: *cells in green; secondary microcavities in blue; and primary microcavity in red*). As a result, the nucleus was often off center and the cells seemed to be polarized and displayed spider-like protrusions spreading away in certain directions (Fig. 1D,E). The ability to relate closely and surround the secondary microcavities appeared to be a preferential property of the osteogenic cells, since it was not normally seen with human gingival fibroblasts; when grown under identical conditions, these fibroblasts displayed a flat and nondescript cytoarchitecture (Fig. 1F). A similar non-descript morphology was seen when osteogenic SBP-DPSC cells were seeded and grown onto smooth scaffold (Fig. 2A–C).

### Expression of osteogenic markers *in vitro*

**Figure 3:**
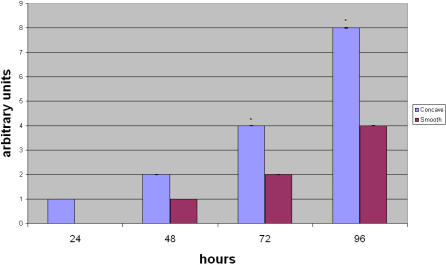
Alkaline phosphatase detection during cell differentiation. The image shows the quantity of ALP during osteoblast differentiation at 24, 48, 72 and 96 hours within the cells cultured on the different surfaces. The data have been rounded to the closest integer value. The error bars are ±SD.*p<0.01. Each experiment was performed in triplicate (n = 3).

To determine whether cell-to-cell scaffold interactions modify phenotypic expression, SBP-DPSC cells were monitored for expression of alkaline phosphatase activity (ALP) at 24, 48, 72 and 96 hours after plating. ALP is an enzyme that is particularly important for osteogenic cells and is required for mineral deposition. Indeed, we observed that ALP was consistently greater in cells plated onto the microcavity-rich substrate (Fig. 3). Gingival fibroblasts did not express ALP.

**Figure 4:**
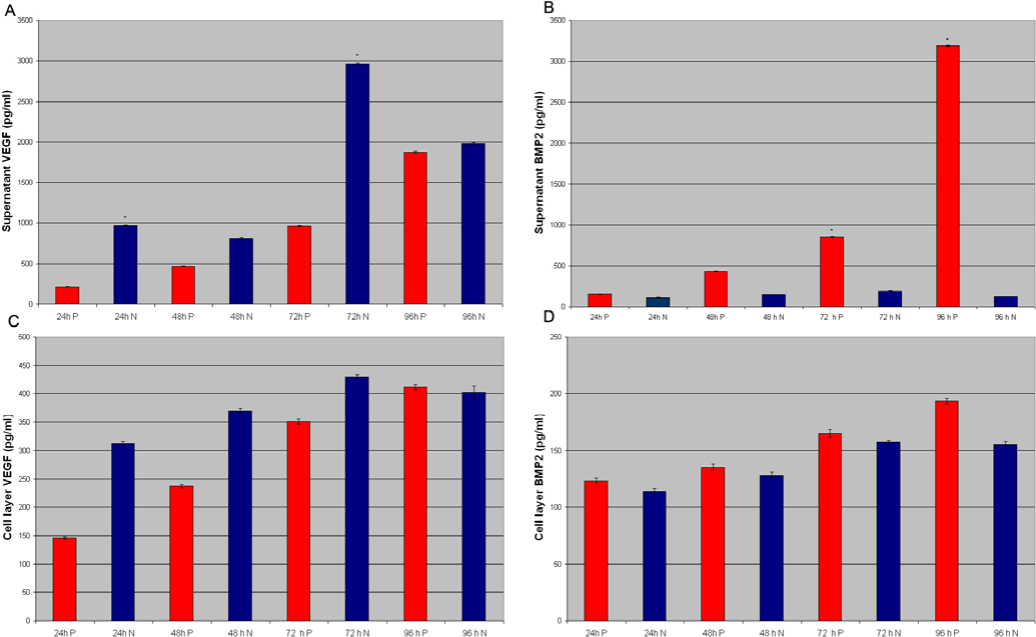
ELISA assays performed on (A) VEGF and (B) BMP2 released within the supernatant by SBP-DPSCs after 24, 48, 72 and 96h from plating on smooth and concave textured surface; ELISA assays performed on (C) VEGF and (D) BMP2 present in the cell layer of SBP-DPSCs after 24, 48, 72 and 96h from plating on smooth and concave texturing. Each experiment was performed in triplicate (n = 3). The error bars are ±SD. *p<0.01. N indicates samples not NMP-treated; P indicates NMP-treated samples.

We then analyzed production and release of BMP-2 and VEGF, factors important for osteogenesis. Greater amounts of VEGF and BMP-2 were present in medium conditioned by cells grown on the microcavity-textured surface, and particularly at 72 and 96 hrs (Fig 4A,B), when compared to amounts present in the medium conditioned by cells on smooth surfaces (Fig 4C, D). Total overall amounts of BMP-2 and VEGF were consistently higher in microcavity rich- than smooth-surface grown cultures. Gingival fibroblasts did not express BMP2 and VEGF.

### *In vivo* bone formation

**Figure 5:**
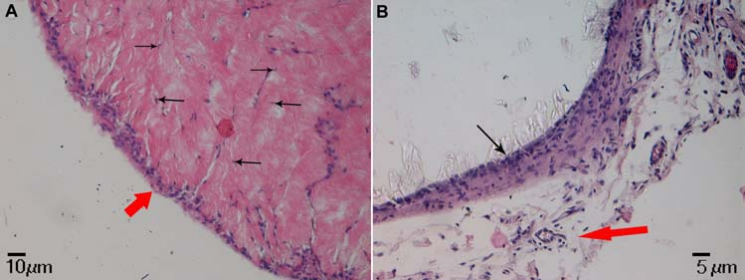
Haematoxilin and Eosin staining. (**A**) *In vivo* sample on concave PLGA scaffold (Calibration Bar = 10 µm). The figures shows that the bone tissue is of great thickness with an evident periosteal layer (red arrow) and containing osteocytes entrapped within the matrix (black arrows). (**B**) *In vivo* sample on smooth PLGA scaffold (Calibration Bar = 5 µm) appeared to be more primitive, thin (black arrow) and not as well developed above a connective loose tissue containing vessels (red arrow).

**Figure 6:**
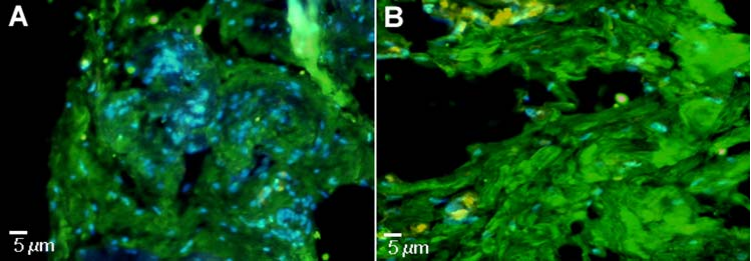
Immunofluorescence for HLA-1 Human FITC (green) (A) *In vivo* concave PLGA scaffold. (**B**) *In vivo* smooth PLGA scaffold. (Calibration Bars = 5 µm). Nuclear staining is obtained with DAPI (blue).

To test the possible influences of surface characteristics on bone formation *in vivo*, SBP-DPSC cells were first grown for about 35 days in monolayer as above and were then seeded onto smooth or concave microtextured scaffolds. Four days later, the colonized scaffolds were grafted into immunocompromised rats, retrieved and analyzed after 30, 45 and 60 days from transplantation. Histological examination indicated that bone tissue had formed on both substrates by day 30 (not shown) and had further remodeled by day 60 into lamellar bone containing osteocytes entrapped within the lamellae when on the microtextured scaffold (Fig. 5A). Tissue present on day 60 on smooth scaffolds appeared to be more primitive and not as well developed (Fig. 5B). The human origin of the bone was confirmed HLA-1 immunofluorescence (Fig. 6).

**Figure 7:**
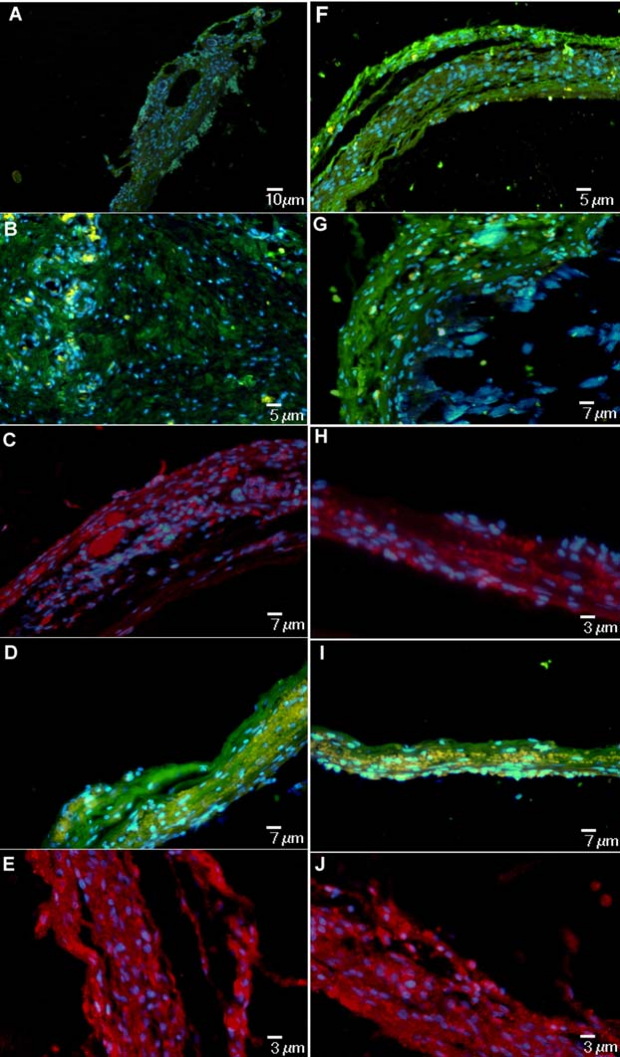
Immunofluorescence confirming the presence of a mineralized extra cellular matrix on concave texturing. The panel shows positivity for Collagen I (**A**) FITC (green) (Calibration Bar = 10 µm), BAP [Bone Alkaline Phosphatase] (**B**) FITC (green) (Calibration Bar = 5 µm), OC [Osteocalcin] (**C**) PE (red) (Calibration Bar = 7 µm), ON [Osteonectin] (**D**) FITC(green) (Calibration Bar = 7 µm) and BSP [Bone Sialoprotein] (**E**) PE (red) (Calibration Bar = 3 µm). The same analysis confirming the presence of a mineralized extra cellular matrix on smooth texturing. The panel shows positivity for Collagen I (**F**) FITC (green) (Calibration Bar = 5 µm), BAP (**G**) FITC (green) (Calibration Bar = 7 µm), OC (**H**) PE (red) (Calibration Bar = 3 µm), ON (**I**) FITC (green) (Calibration Bar = 7 µm) and BSP (**J**) PE (red) (Calibration Bar = 3 µm). Nuclear staining is obtained with DAPI (blue).

Immunohistochemistry showed presence of mineralized extracellular matrix in day 60 microcavity-rich samples that contained bone markers including collagen I, BAP, OC and ON (Fig. 7A,B,C,D). BSP was particularly abundant along the scaffold's edge, indicating possible sites of proliferation and tissue neoformation (Fig. 7E). Performance of cells on the smooth surface was not as vigorous both in terms of immunofluorscence signal intensities and bone tissue thickness (Fig. 7F, G, H, I, J).

## Discussion

In this study we present evidence that dental pulp-derived stromal stem cells behave and respond differently when exposed to microcavity-rich versus smooth PLGA substrates. The concave texturing of the substrate elicits cytoarchitectural responses and adaptation in which the cells appear to favour intimate contacts with the secondary microcavities and cellular polarization. Such behaviour is accompanied by increased release of BMP-2 and VEGF into the culture medium and by higher ALP activity. It is likely that increased release of potent factors such as BMP-2 and VEGF and the higher ALP activity could have significant biological ramifications. By their proven involvement and potency in bone formation and angiogenesis, these factors and enzymatic activity may influence the responses and developmental program of stromal-derived cells via autocrine mechanisms and also influenced by surrounding cells via paracrine pathways. In this likely scenario, increased levels of BMP-2 and VEGF could be responsible for the greater amounts of bone tissue we observe after transplantation of the colonized microcavity-rich scaffold.

The coordinated increase in BMP-2 and VEGF levels suggest that a relationship exists between them during osteogenesis, as previously hypothesized [38]. It is well known that vasculogenesis is of critical importance for bone development and healing [39], [40] and that vessel sprouting is closely linked to bone formation during physiological bone development. This reciprocal interplay is regulated by a BMP-2-VEGF cross-talk. Thus, these factors may induce formation of a bone tissue containing a suitable number of vessels that will ensure a sufficient bone feeding [39]–[41]. Thanks to a high pO_2_ and nutrient concentration, bone then carries on mineralization and maturation process. The VEGF and BMP-2 levels found in our study suggest that the concave-textured surface of the PLGA constructs may facilitate the release of more biologically-relevant and coordinated amounts of these two factors when compared to the amounts released by cultures grown on smooth surfaces.

It is interesting to note that primary micro concavity diameters are similar to those seen in medullary trabecular spaces, while secondary micro concavities resemble niches in the stromal bone marrow, the regulatory depressions for hematopoiesis and ossification. It is conceivable that the structural features of the microcavity-rich surface may have somehow enabled the stromal cells to react more vigorously and favorably then the smooth surface. As stated above, cells on concave texturing are more polygonal in shape with phylopodia-like and lamellipodia-like extensions and appear to have a nuclear polarity that may represent an index of secretion and of other cellular activities, such as matrix formation. These parameters do not characterize the same cell populations plated onto smooth surfaces nor do they characterize gingival fibroblasts plated onto microcavity-rich substrate. It may then be that by mimicking *in vivo* microstructural features and niches, the microcavity-rich substrate may have prodded stromal-derived cells toward osteogenic differentiation and a more vigorous developmental response and outcome.

When a cell sits in a microcavity, when compared with the same cell laying on a flat surface, for the adhesion processes it recruits a higher number of integrins per volumetric unit [5], as it is easily conceivable measuring the area of a circle and the area of an emisphere with the same radius. The higher the number of integrins involved, the higher the number of focal adhesion kinases activated, with an increased exposition to mechanical stresses acting both on the cell and scaffold surface; moreover, as a microwell-plate, the microcavities make easier the interaction of the cell receptors with soluble factors released by cells, influencing their fate [42].

In conclusion, micro-concavities (subtractive pits texturing) elicit superior outcomes in terms of osteodifferentiation, cell maturation and specific protein production, leading to a neoformation of bone tissue of significant thickness. This information could be used to design new scaffolds for bone tissue engineering with textures capable of promoting more effective healing of bone and mineralization defects.

## Materials and Methods

### Dental pulp extraction and digestion

Human dental pulp was extracted from teeth of healthy adult subjects aged 20 to 45 years. Before extraction, each subject was checked for systemic and oral infection or diseases. Only disease-free subjects were selected for pulp collection. Each subject was pre-treated for a week with professional dental hygiene. Before extraction, the dental crown was covered with a 0.3% chlorexidin gel (Forhans, N.Y., USA), for 2 min. Dental pulp was gently removed by means of a dentinal excavator or a Gracey curette, immersed in a digestive solution containing penicillin 100 U/ml, streptomycin 100 µg/ml, 0.6 ml claritromycin 500 µg/ml, 3 mg/ml type I collagenase and 4 mg/ml dispase in PBS, and incubated for 1 h at 37°C. Following this incubation, the digested tissue mixture was filtered through a 70 µm Falcon strainer (Becton & Dickinson, Franklin Lakes, NJ, USA) to obtain a single cell suspension.

### Cell culture

After filtration, the cells were placed in α-MEM culture medium supplemented with 20% FCS, 100 µM 2P-ascorbic acid, 2 mM L-glutamine, 100 U/ml penicillin, 100 µg/ml streptomycin (all purchased from Invitrogen, San Giuliano Milanese, Milan, Italy) and placed in 75 cm^2^ flasks with filtered caps. Flasks were incubated at 37°C in humidified atmosphere containing a 5% CO_2_ and medium was changed twice a week. After cell confluence, the cells were subdivided into new flasks and passaged a total of 10 times. Human gingival fibroblasts were collected during periodontal surgery and cultured as above.

### FACScanning, sorting and differentiation

As previously specified [7], [8], we sorted cells using both morphological traits (high side scatter and low forward scatter) and antigenic criteria (firstly using CD117 and CD34, and then serially using STRO-1 and flk-1). Only cells that expressed all these markers were selected in order to obtain a homogeneous population, called SBP-DPSC.

Briefly, cells were detached using 0.02% EDTA in PBS and pelleted (10 min at 1,000 rpm), washed in 0.1% BSA in 0.1 M PBS at 4°C and incubated in a solution of 1 µl antibody/9 µl 0.1% BSA in 0.1 M PBS. Cells were washed in the same solution once and were processed for sorting (FACsorter, Becton & Dickinson, Franklin Lakes, NJ, USA). The mouse anti-human antibodies CD117 (c-kit), CD34, flk-1 and STRO-1 were from Dr. Torok-Storb through DBA, Segrate, Milan, Italy.

Osteogenic differentiation was achieved as reported by Laino et al. [7], [8]. Briefly, SBP-DPSCs were cultured with 20% FBS for 15 days without passaging, after which cells were cultured with 20% FBS for the rest of the experiment.

To monitor differentiation, the cells were examined using mouse anti-human antibodies to CD44, the transcription factor RUNX-2 (all from Santa Cruz, CA, USA), type I and II BMP receptors (BMPr I–II) and VEGF receptor type II (Flk-1). For RUNX-2 analysis, cells were fixed in 4% paraformaldehyde in 0.1 M PBS and 0.2% Triton X-100 for 30 min at 4°C, washed twice in 0.1% BSA in 1M PBS and then incubated with RUNX-2 antibody.

### Substrate design and cultures

To obtain scaffold with two surface texturing, PLGA 85:15 was carved as membranes (INION, Finland). (A) a smooth surface was obtained using PLGA membrane as sold by the manufacturer; and (B) a concave texturing was obtained treating the membrane with NMP, a chemical agent (by the same manufacturer) that is able to create controlled subtractive pits on the surface following manufacturer's instructions.

Aliquots of 100,000 SBP-DPSCs (CD44^+^/RUNX-2^+^) were gently plated on the scaffolds and cultured for 96 h in order to observe cell-substrate adhesion and protein expression. Gingival fibroblasts were used as control for each substrate. Plating efficiency assays were performed counting free-floating cells 12 hours after plating.

After 96 h scaffolds and cultured cells were transplanted into animals. Experiments were repeated at least four times.

### Scanning electron microscope analysis

After 96 h of culture, cells were fixed in 2.5% glutaraldehyde (EM grade) in 0.1 M phosphate buffer, postfixed in 0.1% OsO_4_ in the same buffered solution for 1 h and, after critical point drying and gold-palladium coating, were observed under a Scanning Electron Microscope (JEOL-6700F, Tokyo, Japan).

### Confocal microscopy

After 96 h of culture, cells were fixed for 15 min at room temperature using 4% paraformaldehyde and permeabilized with 0.2% Triton X-100 in 0.1 PBS, washed twice with 0.1 M PBS for 10 min at room temperature and incubated for 30 min with phalloidin-FITC (Sigma, Milan, Italy), then washed with 0.1 M PBS for 10 min and stained for 2 min with Hoechst Blue (Sigma, Milan, Italy). Cells were washed twice with 0.1 M PBS and then observed on a Zeiss LSM510 confocal microscopy (Carl Zeiss, Göttingen, Germany).

### *In vitro* histochemistry

For alkaline phosphatase (ALP), samples of 100,000 differentiated SBP-DPSCs (CD44+/RUNX-2+) were detached after 24, 48, 72, 96 hours by treatment with PBS/EDTA (0.02%) and centrifuged for 10 min at 140 × g. The pellet was incubated with 1 ml of BMPurple solution (Roche, Segrate, Milan, Italy) for 8 hours in the dark. Supernatant absorbance was measured at 615 nm using a spectrophotometer. As a control, human ginigival fibroblasts cultured on scaffolds were used. Values are expressed as the ratio between sample and BMPurple stock solution. BMPurple solvent was used as blank.

### BMP2 and VEGF ELISA analyses

In order to evaluate BMP-2 and VEGF levels within the cell layer, after 48, 72, 96 hours from plating onto scaffolds, all the cell layer (4×10^6^ cells/sample) of SBP-DPSCs (CD44+/RUNX-2+) were lysed in RIPA buffer (1 mM EDTA, 50 mM Tris HCl pH 7.4, 150 mM NaCl, 0.1% SDS, 0.5% Triton X-100, 0.25% Na-deoxycholate, 1 mM sodium orthovanadate) with 1 µg/ml leupeptin (Sigma), 1 µg/ml pepstatin (Sigma), 1 µg/ml aprotinin (Sigma), and 1 mM PMSF (Sigma). Samples were centrifuged (16,000 × g at 4°C for 20 minutes) and supernatant was precleared on an orbital shaker for 1 h at 4°C with protein-A acrylic beads (Sigma). Following centrifugation (5 min at 12,000 g), protein contents of the supernatants were determined using the Bradford reagent (Bio-Rad, Milan Italy) at 595 nm. Aliquots of 0.5 ml were collected from each sample and analyzed with an ELISA kit for BMP2 or anti-VEGF (R&D, Milan, Italy).

In order to evaluate BMP-2 and VEGF levels in the culture medium, the complete supernatant medium was collected from cultures after 24, 48, 72 and 96 hours from plating (4×10^6^ cells/sample) SBP-DPSCs onto scaffolds. After centrifugation to remove particulates, aliquots of 2 ml were stored at −20°C. After thawing at room temperature, 0.5 ml were collected from aliquots and analyzed with ELISA kit for BMP2 or anti-VEGF (R&D, Milan, Italy). As a control, human ginigival fibroblasts cultured on scaffolds were used.

### *In vivo* transplantation

PLGA membranes colonized with differentiated SBP-DPSCs (CD44^+^/RUNX-2^+^) (4×10^6^ cells/sample) for 96 h were transplanted into the dorsal surface (i.e. subcutaneously) of 10–12 week Wistar rats (Charles River Laboratories Italia S.p.A., Calco, Lecco, Italy). Animals were immunocompromised using Cyclosporine A (Sandimmun, Novartis Pharma S.p.A., Origgio, Varese, Italy) at a dosage of 15 mg/kg body weight, administered 4 h before transplantation and then daily till sacrifice. During the two weeks following surgery, daily dosages were reduced gradually down to 6 mg/kg body weight. Transplants were recovered 30, 45 and 60 days after transplantation. All the above-mentioned procedures were approved by institutional small animal ethics committee.

### Histology and immunofluorescence

Transplants were fixed in 4% paraformaldehyde in PBS for 48 h at 4°C pH 7.4, decalcified for 7 days in 10% EDTA in PBS at RT and then washed in PBS pH 7.4 at 4°C, dehydrated, embedded in paraffin and sectioned (5 µm thick). For histological analysis slides were deparaffinized, hydratated and stained with hematoxylin-eosin and Mallory staining. For immunofluorescence, sections were deparaffinized, hydratated, washed again in 0.1 M PBS, and then blocked in 3% FBS in 0.1 M PBS at room temperature. Mouse anti-human monoclonal antibodies were the following: bone alkaline phosphatase (BAP), osteonectin (US Biological, Swampscott, MA); anti-osteocalcin, anti-osteonectin, anti-BSP and anti- Collagen I (Santa Cruz, CA) were goat anti-human. The secondary antibodies were goat anti-mouse and mouse anti-goat (both FITC conjugated, Santa Cruz). In addition, to evaluate cell distribution inside tissues, DAPI counterstaining was performed. Samples were observed under fluorescence microscopy (X41, Olympus Optical Co. Europe, Hamburg, Germany). The specificity of each antibody was assessed by reacting the above mentioned antibodies with human bone samples from a mandible. Isotype-matched antibodies were used at the same concentrations as negative controls.

### Statistical analysis

Student *t*-test (two-tailed) was used for statistical evaluation. Level of significance was set at p<0.05.
